# Implementing internet-based cognitive behavioural therapy (moodgym) for African students with symptoms of low mood during the COVID-19 pandemic: a qualitative feasibilty study

**DOI:** 10.1186/s12888-024-05542-4

**Published:** 2024-02-01

**Authors:** Joyce M. Ncheka, J. Anitha Menon, E Bethan Davies, Ravi Paul, Sidney O C Mwaba, John Mudenda, Heather Wharrad, Harsa Tak, Cris Glazebrook

**Affiliations:** 1 School of Medicine, Department of Psychiatry, University of Zambia, Lusaka, Zambia; 2 School of Humanities and Social Sciences, Department of Psychology, University of Zambia, Lusaka, Zambia; 3 School of Liberal Studies, University of Petroleum and Energy Sciences, Dehradun, India; 4 Institute of Mental Health, School of Medicine, NIHR MindTech MedTech Co-operative, The University of Nottingham, Nottingham, UK; 5 Lusaka Apex Medical University, Lusaka, Zambia; 6 School of Health Sciences, University of Nottingham, Nottingham, UK; 7 Clinical Neurosciences and Mental Health, School of Medicine, Institute of Mental Health, The University of Nottingham, Nottingham, UK; 8 Rochester Institute of Technology, Liberal Arts Department, Dubai, UAE

**Keywords:** Cognitive behavioral therapy, Low income countries, Qualitative research, COVID-19, Students

## Abstract

**Background:**

Online therapies have been shown to be effective in improving students’ mental health. They are cost-effective and therefore have particular advantages in low-income countries like Zambia where mental health resources are limited. This study aimed to explore the perceived impact of the COVID-19 pandemic and the feasibility of implementing an Internet-Based Cognitive Behavioural Therapy (iCBT) intervention (‘moodgym’) to improve resilience in vulnerable Zambian students.

**Methods:**

The study was a qualitative interview study. Participants identifying as having symptoms of low mood and completing a baseline, online survey (*n* = 620) had the option to volunteer for a semi-structured interview to explore views about their experience of the pandemic and the acceptability and perceived benefits and limitations of using moodgym.

**Results:**

A total of 50 students (*n* = 24 female, *n* = 26 male) participated in the study. One theme with 4 sub-themes, captured the severe emotional and social impact of the COVID-19 pandemic. A second, very strong theme, with 5 sub-themes, reflected the considerable negative effects of the pandemic on the students’ educational experience. This included the challenges of online learning. The third theme, with three subthemes, captured the benefits and acceptability of moodgym, particularly in terms of understanding the relationship between thoughts and feelings and improving academic performance. The fourth theme described the technical difficulties experienced by students in attempting to use moodgym.

**Conclusion:**

COVID-19 caused fear and impacted wellbeing in vulnerable students and severely impaired the quality of students’ educational experience. The findings suggest that moodgym might be a valuable support to students in a low-income country.

## Introduction

In addition to the direct health effects of COVID-19, the efforts to manage the pandemic through lockdowns, suppression of economic activity and closures of schools and universities, have had a detrimental influence on mental health. Young adults are at particular risk, with one study finding that 84% of 21 to 29 year olds reported changes in their mood due to the pandemic, with those with a prior history of depression being less resilient to the negative psychological effects [1]. Mental health impacts are particularly harmful in low and middle-income countries, which typically lack the resources to mitigate these challenges [2]. As with many low-income countries, Zambia has a poorly established mental health infrastructure, with mental health services receiving less than 1% of the national health budget [3]. The pandemic ‘disrupted or halted critical mental health services in 93% of countries worldwide’, with low-income countries in particular being forced to divert resources to shore up inadequate physical health services [4].

University students are a particularly vulnerable sub-group of young people. An online survey found that, when controlled for demographic variables and psychiatric history, French students were more than 50% more likely to experience significant levels of depression and anxiety, and 70% more likely to report high levels of stress compared to non-students during lockdown [5]. A longitudinal Swiss study of university students found symptoms of depression, anxiety, stress and loneliness all worsened significantly during the pandemic, compared to the previous year [6].

The psychological impact of the pandemic on students may be particularly apparent in low-income countries, compounded by economic vulnerability, poor digital infrastructure and lack of access to mental health services. This may contribute to a cycle of disadvantage in terms of economic and health outcomes. Our recent survey of Zambian students found higher levels of anxiety and depression were linked to lower self-efficacy to protect themselves from COVID-19. Furthermore, students scoring above the threshold for possible clinical anxiety (74%) and students with low self-efficacy had significantly lower scores for COVID-19 protective behaviours [7]. Pre-pandemic, higher levels of depressive symptoms have been linked to poorer academic outcomes in university students [8].

The COVID-19 pandemic has created an opportunity to rethink the provision of mental health support in low-income countries by increasing access to psychological therapies such as Cognitive Behavioural Therapy (CBT) [2]. CBT focuses on aiding the individual to recognise dysfunctional, distorted and maladaptive thoughts, which interfere with the individual’s mood and behaviour [9]. A Jordanian study reported CBT to be effective in targeting mental health problems in university students with significantly lowered scores for perceived stress and depressive symptoms. Students randomized to the intervention also reported more use of problem-focused coping strategies [10]. Studies conducted in low-and-middle-income countries have found similar results [11].

Online therapies have been shown to be effective in improving student mental health [12] including iCBT. They are cost-effective [13] and therefore have particular advantages in low-income countries which suffer from a lack of trained mental health professionals [14] and mental health services at the primary care level [3]. Furthermore, digital mental health tools can allow for anonymous and comfortable access in a private setting [15] which may be particularly advantageous for Zambian students, given the lack of mental health services and stigma towards accessing support. Moodgym is one of the most well-known, widely-used and extensively researched iCBT programs [16]. Meta-analysis has shown it to be effective in reducing levels of anxiety and depression [17]. Findings from a qualitative study suggested that an in-house developed, supported iCBT intervention was perceived to help develop skills to manage depression by facilitating self-reflection and was less stigmatizing to access than face-to-face therapy [18]. A meta-synthesis of qualitative studies with a range of populations highlighted the importance of tailoring digital therapies to specific user groups [19]. Moodgym is designed to be acceptable for young people but to our knowledge, no qualitative research has explored the feasibility and acceptability of moodgym for students with depressive symptoms in a low-income country such as Zambia. MRC guidelines stress the importance of considering a range of contextual factors when evaluating complex interventions [20] therefore this study also aimed to understand the experience of Zambian students during the COVID-19 pandemic.

## Methods

### Design

This was a qualitative, interview-based study conducted as part of mixed methods feasibility study.

### Participants and recruitment

The full recruitment process for the wider study is reported in our earlier paper [7]. Participants were recruited between February and July 2021. To be eligible to participate, university students had to be aged ≥ 18 years, in their second year of study or beyond, and self-identify as having experienced at least one key symptom of depression in the previous two weeks, as defined by PHQ-4 [21]. Final-year students were not eligible to participate. Participants who completed the baseline online survey were given the option to volunteer for an interview. The target sample size was 50 students out of the 620 who had completed the baseline survey. The target of 50 interviews is above the median sample size typically reported in qualitative studies in high quality medical journals [22]. Although it has been argued that 12 interviews are sufficient to ensure data saturation with no new themes emerging [23] we wanted to ensure sufficient diversity of experience within our sample.

### Procedure

The information technology services at the University of Zambia (UNZA) and Lusaka Apex Medical University (LAMU) sent invitation emails to all registered students meeting the year of study inclusion criteria (*n* = 21,450). Study aims were explained in the invitation email which directed students who self-identified as having at least one key depressive symptom in the past two weeks (little interest or pleasure in doing things and/or feeling down, depressed, or hopeless) to an online survey hosted on JISC Online Surveys. Students who clicked on the link could then read the participant information sheet and provide online consent by ticking a box. Participants completed the Hospital Anxiety and Depression Scale [24] and questions about their health behaviours during the pandemic [7]. Those who completed the survey were directed to a separate online survey where they could enter their email address. They were then sent a pre-paid, login code and instructions on how to access moodgym. At the same time, they were also given the opportunity to participate in a semi-structured interview to explore their mental health, the impact of COVID-19 pandemic, and their experiences of using moodgym. Volunteers for the interview study could not be linked to their survey data in order to preserve the anonymity of the survey.

Consenting participants were contacted via email to take part in a semi-structured interview with a Zambian researcher (JMN). Interviews were conducted between July and November 2021, a minimum 10 weeks after participants had received the moodgym login. Participants were given the option to be interviewed via telephone, online or face-to-face on the university campus. The interviews were conducted in English and were recorded using a mobile phone, with the data roaming function disabled. The audio files were transferred to a password-protected file on a dedicated laptop for the study. These interviews were transcribed verbatim by one of two researchers (HT or JMN) identified only by participant number and with any identifiers removed. Audio recordings were subsequently erased after checking the transcriptions. Students who participated in the interview were given a shopping voucher worth ZMK150 (Zambian Kwacha; approximately £6.00) to thank them for their participation.

#### Interview schedule

The interview schedule consisted of open-ended questions inviting students to discuss their experience of the COVID-19 pandemic and their use and perceptions of moodgym. Additional questions on COVID-19 health protective behaviours and perceptions of COVID-19 vaccination have been analysed in a previous paper [7]. Interviews lasted an average of 25 min.

### Ethical approval

Ethical approval was obtained from the University of Zambia Biomedical Research Ethics Committee (UNZABREC- 1296–2020) and the National Health Research Authority (NHRA). Permission was also sought from the management of the University of Zambia and Lusaka Apex Medical University. Psychological support was made available to any interviewees who were judged to be experiencing concerning levels of emotional difficulties.

### Data analysis

Data were analysed inductively using thematic analysis [25]. The transcribed interviews were read and reread, noting down initial codes. Important features of the data relevant to the research questions were identified and generated into codes. Significant broader patterns of meaning in the codes and data were identified and grouped into themes by two researchers (JMN and HT) and given meaningful titles. The themes were related back to the research questions to ensure they were appropriate. These themes were reviewed and modified by a third experienced qualitative researcher (CG).

## Results

Out of 186 participants who consented to take part in interviews, 108 were sent invitation emails and the first 50 to respond were interviewed. Of these, 26 (52%) were male and 24 (48%) were female. Demographic characteristics of the sample are shown in Table 1.

**Table 1:** Demographic characteristics of the sample

**Gender**	
Male Female	26 (52%) 24 (48%)
**Year of study**	
Year 2	13 (26%)
Year 3	19 (38%)
Year 4 and beyond	18 (36%)
**Major of study**	
Medicine	10 (20%)
Pharmacy	1 (2%)
Engineering	6 (12%)
Economics	2 (10%)
Computer Sciences/Information and communication technology	5 (4%)
Education/Special education	9 (18%)
Biomedical Sciences	5 (10%)
Diagnostic Radiography	2 (4%)
Nursing/Midwifery	3 (6%)
Demography and gender studies	7 (14%)

Four overarching themes with 12 subthemes were identified: (1) COVID-19 has caused stress and impacted wellbeing; (2) COVID-19 has impaired the quality of students’ educational experience; (3) Moodgym helped to support mental wellbeing; and (4) Students experienced challenges using moodgym (Table 2).

**Table 2:** Themes and subthemes

Theme	Subtheme
1. COVID-19 has caused stress and impacted wellbeing	1a Experience of elevated fear and uncertainty. 1b Personal experience of COVID-19 drives distress. 1c COVID-19 has caused financial stresses 1d. Social isolation is an “emotional drain”
2. COVID-19 has impaired the quality of students’ educational experience	2a. Technical and practical issues disrupted online learning 2b. Reduction in academic motivation and performance 2c. Additional pressure of work 2d. Needed guidance from lecturers 2e. Learning depends on group discussions
3. Moodgym helped to support mental wellbeing	3a. Moodgym improved understanding of how thoughts influence feelings 3b. Moodgym has helped to improve social interactions. 3c. Moodgym has academic benefits
4. Students experienced some challenges with moodgym	

### COVID-19 has caused stress and impacted wellbeing

This overarching theme captures the psychosocial impacts of the COVID-19 pandemic for Zambian students and the resulting economic and health uncertainties.

#### Elevated fear and uncertainty

This sub-theme reflects a general feeling of fear associated with the pandemic.

“We are not even sure how the future will be… mostly it is just anxiety about the future” (P2, F).

This excessive fear was often driven by rumour and the media.

“… these things were put on social media. We were seeing large numbers of people dying” (P12, M).

Consequently, there was a tendency to catastrophise due to fear and uncertainty.

“Things that never used to get to you are now getting to you, I don’t know how to put it but…. Because I have been hearing a lot of bad news, people are dying of COVID. It just gets to your brain and you are thinking, ‘what if today is my last day’ or ‘what if today is my parents last day’. You just get into depression when you think of all these people who are dying, people who are close to you, why is it happening?” (P3, F).

#### Personal experience of COVID-19 drives distress

This sub-theme captures students’ direct experiences with COVID-19 infections and its implications for themselves and their families. Many of the students had themselves experienced serious illness or death of friends, family and public figures such as doctors, lecturers or religious leaders occurring as a result of the COVID-19 infection.

“I did suffer from COVID myself… it was a really a bad experience… I was very sick. I was in hospital for a week… I was on oxygen… my oxygen saturation was 60” (P20, M).

“It’s been devastating at multiple levels… I’ve seen at least three of my closest family members die of the pandemic” (P18, M).

This was particularly challenging for students on clinical courses who recognised their own vulnerability.

“I had this friend of mine from church, she was a doctor… she passed away. And we were really scared being fourth year… so I started thinking that even us when we become maybe interns we will be exposed to a lot of diseases, who knows maybe you can get sick of COVID-19 and all those thoughts… this started moving around in my mind (P38, M).

#### COVID-19 has caused financial stresses

At the beginning of the COVID-19 pandemic, measures were put in place such as lockdown, restrictions in movement and staying home, which could have affected the daily routines for most people. This led to parents losing jobs, loss of family businesses and loss of income which affected students’ financial security as many depended on parents for financial support. The financial problems also led to strained relationships.

“Financially… mostly, my Dad was laid off from his job… we are a family of 10… my Dad has financial problems and Dad and Mum’s relationship is not good at the moment (P11, F).

Some students were involved in small businesses to support their education and livelihood and reported losing them as a result of the pandemic which worsened their financial problems.

“The difficult part was… some of us find some source of income out there where you could go and find some money. Now… just staying indoors, you find that you want some bundles for e-learning and just some simple needs that we require, so it is hard to stay indoors” (P12, M).

#### Social isolation is an “emotional drain”

Whilst the implementation of the lockdown was an effective measure to protect the physiological health of individuals, as it prevented and reduced the infectious spread of the COVID-19 virus, it was perceived to fuel negative psychological states in Zambian University students. Access to activities that supported wellbeing was restricted and over half of students expressed concern about the effects of social isolation.

“Before COVID, we would organise games… football… I am a soccer fan. We had time playing soccer with friends. Now, most things are restricted, we can’t get together and have that communal experience with friends as we used to, playing soccer and other activities, because of the fear of spreading or contracting COVID” (P8, M).

Some students complained about the inability to physically associate with friends because of the restrictions on movements.

“We don’t get to meet a lot of friends because we have to be cautious of the pandemic all the time. That’s one of the challenges and sometimes I want to hug my friends because that’s the only consolation now, because we are isolating and there is just that emotional drain isolating yourself. You need to be with friend when you see them after a long time, you get excited and want to hug them and it’s a challenge” (P43. M).

Students also expressed feeling trapped at home following the order given by health experts arguing people stay home which could have affected their mental wellbeing.

“It doesn’t feel great being home almost all the time, I feel trapped. Yes, my social life has disappeared completely. I don’t do anything I used to do before corona, I can’t go out for lunch with my friends because we all do not want to get the virus” (P1, F).

The loss of daily activities and schedules due to the lockdown was perceived to have increased psychological distress.

“I have enough time to like over think much and worry more, my anxiety levels too are high” (P1, F).

Students further expressed feelings of loneliness and concerns about the loss of relationships due to the lockdown.

“We have lost that sense of togetherness and that relationship… you feel like you are alone” (P12, M).

### COVID-19 has impaired the quality of students’ educational experience

This was a very strong theme with nearly nine out of every 10 students reporting experiencing distressing disruptions to their education as a result of COVID-19 and associated restrictions.

“All these challenges have really affected my academics at school and after the indefinite closure, it really impacted me emotionally… you are expecting something like finishing school early and COVID-19 has come and you know… affected everything.” (P14, M).

Female students appeared more negatively impacted by university closures as their families expected them to take part in the household daily chores.

“I will talk about the chores, of course in a home as a girl child you are supposed to be cleaning… cooking. Because I am a girl child, I am supposed to be cooking and cleaning, no one even considers the fact that I am supposed to be learning.” (P23, F).

#### Technical and practical issues disrupted online learning

During the peak of the COVID-19 pandemic, universities around the globe moved from physical classes to online learning overnight, following the order from Governments to have citizens stay home as a preventive measure to reduce COVID-19 infections. Several technical and practical issues prevented students from adequately adapting to their new learning environment. Numerous students expressed problems with the lack of internet, technological gadgets, and networks required for online learning. This made it difficult for students to be fully involved in their academic work.

“… the phone was a challenge and when I tried to access another phone, I still had issues with finding bundles. And… also you find that amidst some classes, you find that the network connection is poor… even giving problems when answering some assignments. I remember one time, I missed a test because of the same bad connections”. (P11, F).

Many students did not have the resources needed to access online teaching.

“I don’t have a laptop yet; one needs a laptop for this course. When we were in school, I used the school computers but at home, it has been a problem. I have been using my mum’s phone for assignments and classes. It is mainly the issue of the laptop; it has made the online learning difficult… all assignments we get… they need a laptop” (P48. M).

The cost of buying data bundles was also a strong recurring issue and the poor quality of the network coverage.

“It has mainly been the issue of bundles for online learning…… Online learning is difficult because of bundles and sometimes even if there is money for bundles… network might be bad.” (P27, M).

#### Reduction in academic motivation and performance

These technical and practical issues manifested as a reduction in academic motivation, which was seen in many students who presented with feelings of fatigue, procrastination, and hopelessness.

“I am not productive at all… I just want to stay in bed and do nothing. I stay in bed for as long as I am feeling bad. I miss classes and if it’s online, I do not join” (P13, F).

The effects of this psychological distress can be seen, as many students reported a decline in academic performance due to the pandemic.

“I haven’t done most of the assessments this year, the only ones I wrote, I have not yet picked up the results. But I can talk based on my first- and second-year results. In first year, my highest grade was an A, I would get an A, B+…but nowadays, despite us having the e-learning platform, my grades are really… in between B and C (P10, F).

This student attributed the lack of motivation to online learning and its lack of a monitoring mechanism.

“…. you are forced to cheat because one no one is there to watch you on an online test… there is no need to study and you try to motivate yourself not to cheat but you end up with the book and the foundation there in not going to be strong because there…” (P23, F).

This poor academic performance may have further contributed to reinforcing negative cognitive states in students leading to pressure of work.

‘Yes, obviously you have to be in the right frame of mind to study and keep up with school, it’s all about the mind… whatever you do depends on your state of mind” (P20, M).

#### Pressure of work

Participants reported feeling under significantly increased academic pressure as a result of the disruption caused by the pandemic.

“I would say… it is not really managing…because there is too much pressure… not enough time to catch up” (P20, M).

University closures led to the piling up of work making it difficult for students to keep up with it.

“I think most of our lecturers didn’t think we would close for a long time, so when we opened, they rushed us through the topics so much… squeezed everything in so we can write exams. It was really so hard to catch up… so much stress… so much pressure to try to get good grades. And this year has also been the same” (P13, F).

Other students could not do their university work whilst at home and opted to wait until schools opened.

“When I went to school, that’s when I wanted to cover up all I missed… there was stress… a lot of work in a short time…” (P29, M).

Due to a lack of time to learn before the end of the academic year, students felt that they might be examined on topics missed due to technical issues with online learning.

Other students recorded online lectures to listen to them later, and the recordings were too many to catch up with leading to the pressure of work.

“I could record the lectures and listen to them later and I could go back where I was not sure. But the recordings became too many to keep up with” (P33. F).

#### Needed guidance from lecturers

The lack of personal contact with teaching staff was strongly perceived to have negatively impacted the quality of students’ learning. Some struggled to understand content when the material was delivered remotely.

“My studies to be honest have been so affected. It’s easy when you are seeing someone, you get to understand more as I can ask when am seeing my lectures face to face… Currently the only way I can contact them is via phone which is a challenge again because sometimes I don’t even have credit to call them” (P7, M).

Others found it difficult to organize their work without direct supervision from the teaching staff.

“So there isn’t that connection between us and them so it’s really… really difficult and also us having to manage our own time without supervision from our lecturers… it’s difficult to manage our time” (P42, F).

#### Learning depends on group discussions

The online learning and university closures put in place to reduce human contact and possible COVID-19 infection meant students could not meet for group discussions with peers, hence reducing social support from peers and impacting learning.

“… most of us are used, when we are given an assignment or maybe classwork, you meet with your friends to discuss… you teach one another… there was nothing like that, just be given assignment and there you are alone trying to solve it by yourself’ (P12, M).

The lack of peer support was felt particularly strongly by students who struggled with lone working.

“I am a person who finds reading by myself difficult, I depend on group discussions… but this year, we started off mostly at home and then when we got back here, gatherings are discouraged… so it’s a big blow on my part” (P41, M).

### Moodgym helped to support mental wellbeing

Students found moodgym to be valuable for improving their mental wellbeing, especially for those who lacked psychological support.

“I am sure that’s why you introduced this moodgym to try and help us psychologically, because the moment we just heard that people were dying…” (P12, M).

Most students who accessed moodgym claimed that it benefitted their mental health and improved their understanding of how thoughts influence feelings.

#### Moodgym improved understanding of how thoughts influence feelings

The majority of students appreciated the insight that moodgym had given them into the relationship between their thoughts and their feelings and the strategies that could be used to challenge their thoughts and thus improve their feelings.

“It helped me to realise that we are in control of our feelings. In as much as something happens, it is temporary. It is up to me to decide whether that should affect me permanently” (P6, F).

“We are all in control of our situation, no one can do it for us… people say I feel bad, there is no such thing as feeling bad… you make yourself feel bad. You can make yourself feel good” (P46, F).

Students appreciated the need to practice gaining control of their thoughts.

“If you think of something bad, you feel bad as well. You think of a good thought, you feel good too. That’s something I have been trying to practise, but with COVID, it has become very difficult to practice. Being home, there are so many people, you get on each other’s nerves, and it’s difficult to practise” (P3, F).

#### Moodgym has helped to improve social interactions

Participants found moodgym to have social benefits as well. Some students reported that they had a negative view of others which made it hard for them to make friends. Students were also seen as possessing an increased understanding of others.

“Yes, there were [benefits], especially socially… I was one of those who quickly jump to conclusion, hard to make friends, you always feel they are thinking ill of you” (P2, F).

It also helped to break down social barriers.

“I was that person who was locked up. I was really… really locked up I wasn’t that much open to the world. Now at least I do talk to people I have close friends now more than I was before I was on this thing [moodgym]” (P38, M).

A few students specifically mentioned that the change in their thinking had helped to improve their self-esteem which had benefits for social interactions.

“I feel good about myself, previously I wanted approval from others to feel good… not anymore”. (P48, M).

Furthermore, an increased understanding of their own behaviour and feelings helped them develop more sensitive and empathetic interactions.

“I used to judge people a lot based on their actions… affected the way I felt, now I am able to handle different people without arguing, I am understanding people more” (P33, F).

#### Moodgym has academic benefits

The understanding of how thoughts influence feelings further encouraged improved academic resilience in many students among the students who had used moodgym. Around a quarter of students attributed academic improvement to using moodgym.

“After it (moodgym) taught me to be positive, put in effort, I started putting in effort. After I completed moodgym, I still was not selected for the course I wanted [quota], I started doing a course online, animal welfare. I was very much focused on this course, I did really great, and I got a certificate” (P4, F).

Some students attributed their improved academic performance to their improvement in mental wellbeing.

“… when I am low, I will not have interest in school but if I am happy… I am mentally okay, then I can handle my work properly without anxiety… without worrying about what grade I will get” (P40, M).

### Students experienced some challenges with moodgym

This was a deductive theme in which researchers specifically looked for limitations of moodgym. This was fairly weak theme with a few students reporting technical and access difficulties using moodgym; mainly related to creating a user account and login.

“I didn’t manage to register for an account, I kept trying but failed” (P46, F).

“I never used the moodgym… aah website… because every time I would try to log in it would ask me to pay (P37, F).

One student found moodgym to be culturally inappropriate for students in the Zambian context.

“… the scenarios were a bit too westernized… scenarios… yes, the scenario… the context that was being used there was a bit western… we see most of those things happening in the US and stuff” (P21, M).

Another student found it difficult to practise using moodgym in the crowded family home.

“…. but with COVID, it has become very difficult to practice (moodgym exercises). Being home, there are so many people, you get on each other’s nerves, and it’s difficult to practise” (P3, F).

## Discussion

This qualitative feasibility study generated two strong themes capturing the experience of COVID-19 in relation to emotional wellbeing and academic experience. One overarching theme, with three sub-themes, identified the ways in which moodgym helped to support academic performance and mental and social wellbeing. A final, weaker, deductive theme captured technical difficulties associated with using moodgym in that setting.

Theme 1 captured powerfully the impact of COVID-19 on the young people in this study. Many had distressing personal experiences of COVID-19. Although the published mortality figures for COVID-19 suggest that Zambia was less affected than many higher income countries, it has been argued that death rates were substantially under-reported in lower income countries [2]. This is reflected in the many personal accounts of loss found in this study.

Most students interviewed were extremely fearful for themselves and for their families. Almost three-quarters of the group from which this sample was drawn were found to have levels of anxiety above the level for possible clinical disorder [7]. In addition to their personal experiences, it is likely that their sense of vulnerability was heighted by a lack of credible public health information. Our previous research highlighted that even in this well-educated group, participants were susceptible to harmful myths about COVID-19 and associated vaccination programme [7].

Low-income countries were particularly vulnerable to the economic impact of the pandemic and many students interviewed in this study had expressed concerns about the financial difficulties experienced by themselves and their families. Our findings are consistent with a survey carried out in Ethiopia, Malawi, Nigeria and Uganda which showed that 77% of participants came from households that experienced financial struggles during the pandemic [26]. The majority of students felt that the social isolation driven by the pandemic heightened their emotional distress. It is likely young people are particularly vulnerable to the loss of peer support and activities that support mental health. An online survey to identify the perceived consequences of social isolation on staff and their work and on students and their studies at universities reported that the abrupt enforcement of social isolation due to COVID-19 restrictions led to staff and students facing problems of lack of social interactions, motivation, and mental health problems that included anxiety [27].

The second, very strong theme, contributed to by 88% of the participants, captured the catastrophic impact of the pandemic on students’ educational experience. Learning had abruptly changed from a classroom setting to online delivery making it difficult for students to adapt. In a low-income economy like Zambia, most students do not own devices such as laptops or smartphones and lack access to good internet connectivity, making it difficult for students to adapt to the new learning environment. A study conducted in Jordan also showed that the lockdown experienced during the COVID-19 pandemic interrupted learning. Students reported that the challenges with online learning, including internet connectivity issues and lack of personal devices for attending the online classes led to feelings of anxiety [28].

Most students felt that their academic motivation and performance declined greatly. This is supported by a study with Bangladeshi students that found students did not feel motivated to engage in academic activities at home as they did not follow any schedule [29]. Students in our study felt under more pressure due to online learning and this supported by other studies. University students in Western India perceived online learning increased their workload [30]. Focus groups with UK students found that some students felt that online learning increased workload and perceived that university staff underestimated the additional cognitive and emotional demands of online learning [31].

Most participants described lack of contact with the teaching staff to negatively impact the quality of their learning, as it was difficult for them to understand the lessons delivered online. Students reported that lessons were easier to understand when there were interactions with lecturers in a face-to-face physical class. Quality of classroom interactions has been shown to be particularly important to wellbeing where students are struggling with online learning [32]. Although a Canadian study also found students to be less engaged with online learning during the pandemic, techniques such as using a synchronous chat function increased students’ sense of belonging and encouraged engagement [33]. Students in our study with little experience of online learning and limited access to specialist technology were at a particular disadvantage.

Most students preferred working in groups, but the restrictions put in place by the health sector to reduce human contact in an attempt to combat the spread of COVID-19 infections reduced social activities among students. This led to non-availability of group discussions with classmates that provided academic support. Group discussions were preferred by many students compared to studying alone. This is supported by a Swedish study which found 97% of students interviewed perceived that working in a group assisted learning, academic knowledge and collaborative abilities,

Compared to lone working [34].

Thus, the context in which moodgym was delivered was characterised by emotional and economic distress as well as multiple concerns about the impact of the pandemic on their education. iCBT delivered following heightened threat such as armed combat may be less acceptable and less effective [35].

However, participants in this study reported that they found moodgym helpful in many ways including, improved mental wellbeing, social interaction, educational experience and self-esteem.

Improvements were facilitated by participants’ understanding of the links between thoughts and feelings and the importance of managing harmful thoughts. This accords with a Swedish qualitative study with vulnerable participants (Arabic speaking refugees and immigrants) which identified “New

ways of thinking and knowing” as a theme [36]. Other studies have suggested that the introduction of self-guided iCBT may be acceptable and effective for university students in non-western cultures during a pandemic, with high uptake and reduction in anxiety and depressive symptoms [37]. Some participants noted that an enhanced sense of self-worth had helped to improve social interactions. Participants described a more sympathetic view of others which helped in making new friends, understanding of others without jumping to negative conclusions and having empathy for other students’ concerns. This positive impact on social interactions following a time of heightened threat is supported by a study with earthquake victims which found that iCBT was associated with improved perceptions of emotion-based social support [38].

The negative impact of COVID-19 on students’ educational experience was a key concern for students so it is noteworthy that many participants in our study reported that moodgym improved academic resilience and academic motivation. There has been little research on the impact of digital mental health interventions on students’ academic functioning [12]. However, a study conducted in United Arab Emirate (UAE) reported improved academic performance following the use of moodgym [39].

A few participants experienced challenges using moodgym. They mainly reported having experienced technical difficulties in the use of moodgym which involved failure to create user accounts. Others managed to create accounts but were too busy with academic work and other activities to use. A study conducted in Netherlands also found that participants reported being busy. Participants in that study preferred more personal contact as iCBT did not create the pressure to complete subsequent sessions. They would also have liked to express their thoughts through talking to someone as opposed to writing them down [40]. Interestingly, students in the present study did not express a preference for face-to-face therapy, perhaps because of the limited availability of CBT in Zambia. ICBT applications are easily available, flexible and moderately anonymous. This makes them particularly suitable for university students [41]. For example, iCBT was found to be associated with better compliance in people diagnosed with MDD, with the iCBT group completing significantly more sessions than those in the in-person group [42]. Mindfulness-based CBT (MCBT) was developed initially to support people recovering from depression and prevent relapse. It combines elements of CBT with mindfulness meditation exercises and has been shown to have comparable efficacy to CBT for the treatment of depression [43]. MCBT is available as an online therapy and further research could explore its acceptability in a developing country such as Zambia.

Since one student felt that moodgym was too westernized and inappropriate for Zambian university students, further research could also evaluate appropriate cultural adaptations. A recent meta-analysis found iCBT to significantly reduce anxiety in university students with a medium effect size but none of the 15 randomised controlled trials were conducted in low-income countries [44]. The findings from this qualitative study suggest that one particular benefit for vulnerable students in a time of crisis is the impact of CBT on social relationships which suggests that a future randomized controlled trial of the intervention in low-income countries should include perceived social support as an outcome. Our findings also suggest that students from a low-income country such as Zambia may need technical and financial support to access iCBT. Informed by this qualitative study and the analysis of our pre-post quantitative data, a future implementation study could evaluate the adaptations necessary for the successful introduction of iCBT into student mental health services in sub-Saharan African.

The strengths of the current study are that it had a large sample of vulnerable students (*N* = 50) from a low-income country who had been offered the opportunity to use moodgym during the height of the pandemic. Additionally, this study had a high proportion of males at 52% recruited as they are usually under-represented in this type of study [45]. A potential limitation of the study is that interviews were conducted over the phone and so the interviewer could not directly observe the reactions of the participants. Furthermore, since the survey was anonymous, we could not use details of depression scores or past help seeking to characterize the sample. However, to our knowledge, this is the first research to explore the feasibility of implementing moodgym with vulnerable populations/students during a pandemic using a qualitative approach.

## Conclusion

COVID-19 impacted wellbeing and severely impaired the quality of student’ educational experience, Moodgym was seen to be a valuable support during the pandemic for vulnerable students by fostering and sustaining social relationships and by helping them to understand and control negative thoughts. Following these results, it is suggested that universities put in place mechanisms to identify struggling students in order to build academic and emotional resilience. The results of the study suggest that there is a need for mental health interventions during a time of crisis such as a pandemic and that moodgym is a feasible and acceptable intervention for students in a low-income country with limited mental health resources.

## Data Availability

Data are available from the corresponding author on reasonable request.

## Abbreviations

CBT: Cognitive Behavioural Therapy
iCBT: Internet delivered Cognitive Behavioural Therapy
UNZA: University of Zambia
LAMU: Lusaka Apex Medical University
UNZABREC: University of Zambia Biomedical Research ethics committee
NHRA: National Health Research Authority
UAE: United Arab Emirates
GPA: Grade point average
CICT: Centre for Information Communication Technologies
